# Divergent mycorrhizal strategies and nutritional modes in the East Asian orchid genus *Ephippianthus*

**DOI:** 10.1007/s00572-026-01285-0

**Published:** 2026-07-02

**Authors:** Kenji Suetsugu, Hidehito Okada

**Affiliations:** 1 Department of Biology, Graduate School of Science, Kobe University, 1-1 Rokkodai, Nada-ku, Kobe, 657-8501 Hyogo Japan; 2 Institute for Advanced Research, Kobe University, 1-1 Rokkodai, Nada-ku, Kobe, 657-8501 Hyogo Japan

**Keywords:** Calypsoinae, Cyphellaceae, DNA metabarcoding, Orchidaceae, Partial mycoheterotrophy, Rhizoctonia, Saprotrophic fungi, Stable isotope analysis

## Abstract

Orchids provide an ideal system for examining the evolution of heterotrophy because all species depend on fungal carbon during germination, whereas adult nutritional modes range from autotrophy to full mycoheterotrophy. The subtribe Calypsoinae illustrates this diversity through repeated shifts in fungal partners and nutritional strategies. Recent phylogenomic analyses place the *Dactylostalix*–*Ephippianthus* clade as sister to the *Corallorhiza* clade, which includes several heterotrophic lineages, raising questions about adult nutrition in *Ephippianthus*. Here, we combined fungal metabarcoding with δ¹³C and δ¹⁵N analyses to investigate the physiological ecology of the two known *Ephippianthus* species, which are restricted to Japan and the adjacent Russian Far East. We found that *Ephippianthus schmidtii* was primarily associated with Tulasnellaceae (Cantharellales). Its δ¹³C and δ¹⁵N values showed no significant positive enrichment relative to co-occurring autotrophic plants, providing no evidence for substantial fungal nutrient gain based on these isotope markers. By contrast, *Ephippianthus sawadanus* was mainly associated with saprotrophic Cyphellaceae (Agaricales), representing, to our knowledge, the first documented case in which a member of this family was detected as the dominant mycorrhizal associate of a green orchid. Moreover, *E. sawadanus* exhibited elevated δ¹³C and δ¹⁵N values relative to autotrophic plants, consistent with substantial fungal contributions to its carbon and nitrogen budgets. Together, these findings reveal divergent mycorrhizal strategies and nutritional modes in the East Asian orchid genus *Ephippianthus*.

**Supplementary Information:**

The online version contains supplementary material available at 10.1007/s00572-026-01285-0.

## Introduction

Orchidaceae is one of the largest angiosperm families and provides a model system for examining the evolution of mycoheterotrophy. Orchid seeds lack endosperm and germinate only after colonization by mycorrhizal fungi, which provide carbon and mineral nutrients (Merckx 2013). Although many orchids later develop photosynthetic leaves and become predominantly autotrophic, others retain partial dependence on fungal carbon as adults, and more than 250 species are fully mycoheterotrophic throughout their life cycle (Merckx 2013; Jacquemyn and Merckx 2019).

Green orchids usually associate with rhizoctonia fungi, a polyphyletic group of saprotrophic or endophytic basidiomycetes in Ceratobasidiaceae, Tulasnellaceae, and Serendipitaceae (Dearnaley et al. 2012; Rasmussen and Rasmussen 2014). By contrast, orchids with stronger fungal dependence often associate with ectomycorrhizal fungi in families such as Sebacinaceae, Thelephoraceae, and Russulaceae, or with saprotrophic non-rhizoctonia fungi in families such as Mycenaceae and Psathyrellaceae (Taylor and Bruns 1997; Bidartondo et al. 2004; Julou et al. 2005; Ogura-Tsujita et al. 2009; Suetsugu et al. 2022). Stable isotope analysis is useful for detecting fungal nutrient gain, as mycoheterotrophic plants often have elevated δ¹³C, δ¹⁵N, and δ²H values relative to autotrophic references (Gebauer and Meyer 2003; Hynson et al. 2013). However, δ¹³C may be less diagnostic in rhizoctonia-associated orchids, which can show little or no ¹³C enrichment despite fungal carbon gain (Gebauer et al. 2016; Gomes et al. 2023; Zahn et al. 2023).

The subtribe Calypsoinae (Epidendroideae) comprises approximately 80 species in 13 genera and shows marked diversity in vegetative morphology, fungal associations, and nutritional strategies (Barrett et al. 2025). Recent phylogenomic analyses revised the evolutionary framework of Calypsoinae by placing the *Dactylostalix*–*Ephippianthus* clade as sister to the *Corallorhiza* clade, which includes *Aplectrum*, *Cremastra*, *Govenia*, *Oreorchis*, *Kitigorchis*, and *Corallorhiza* (Barrett et al. 2025). This placement is important for understanding the evolution of mycoheterotrophy.

Several members of the *Corallorhiza* clade show strong partial or full mycoheterotrophy and associate with ectomycorrhizal or saprotrophic non-rhizoctonia fungi. For example, the species treated as *Oreorchis indica* in Suetsugu et al. (2021), now assignable to *Kitigorchis*, associates with the ectomycorrhizal genus *Tomentella* and shows pronounced heterotrophy. Species of *Corallorhiza* also associate with ectomycorrhizal fungi in Thelephoraceae or Russulaceae and show strong heterotrophic tendencies (Barrett et al. 2010; Freudenstein and Barrett 2014; Suetsugu et al. 2021). By contrast, saprotrophic Psathyrellaceae associations occur in some *Cremastra* species and coralloid-rhizome-bearing *Oreorchis patens*, where they are linked to increased fungal carbon gain (Yagame et al. 2021; Zahn et al. 2022; Suetsugu et al. 2022, 2026; Suetsugu and Okada 2025a, b). Although *Dactylostalix ringens* and *D. uniflora* are highly specific to putatively saprotrophic *Oliveonia* fungi (Auriculariales), their carbon isotope signatures provide no evidence of detectable fungal carbon gain (Suetsugu and Okada 2025c). However, cryptic partial mycoheterotrophy cannot be excluded without δ²H and δ¹⁸O data.

The fungal partners and adult nutritional modes of *Ephippianthus* remain uncharacterized. Although these species occur in shaded forest-floor habitats that may favor partial mycoheterotrophy, their slender rhizomes differ from the coralloid rhizomes associated with strong fungal dependence in several other Calypsoinae (Yagame et al. 2021; Zahn et al. 2022; Suetsugu et al. 2022; Suetsugu and Okada 2025a, b, d). Determining the fungal partners and nutritional strategies of *Ephippianthus* is therefore essential for understanding the evolution of mycorrhizal shifts and fungal dependence within Calypsoinae.

Here, we examined the two known *Ephippianthus* species to determine whether they associate with rhizoctonia fungi or alternative fungal lineages. We combined fungal metabarcoding of mycorrhizal rhizomes with δ¹³C and δ¹⁵N analyses of leaves and co-occurring autotrophic reference plants. Specifically, we asked whether the two species differ in dominant fungal partners and whether their δ¹³C and δ¹⁵N values indicate partial mycoheterotrophy. Because δ²H values were not analyzed, we interpret the absence of ¹³C and ¹⁵N enrichment as no detectable fungal nutrient gain based on the available markers, rather than definitive evidence against partial mycoheterotrophy.

## Materials and methods

### Study sites and sampling scheme

**Fig. 1:**
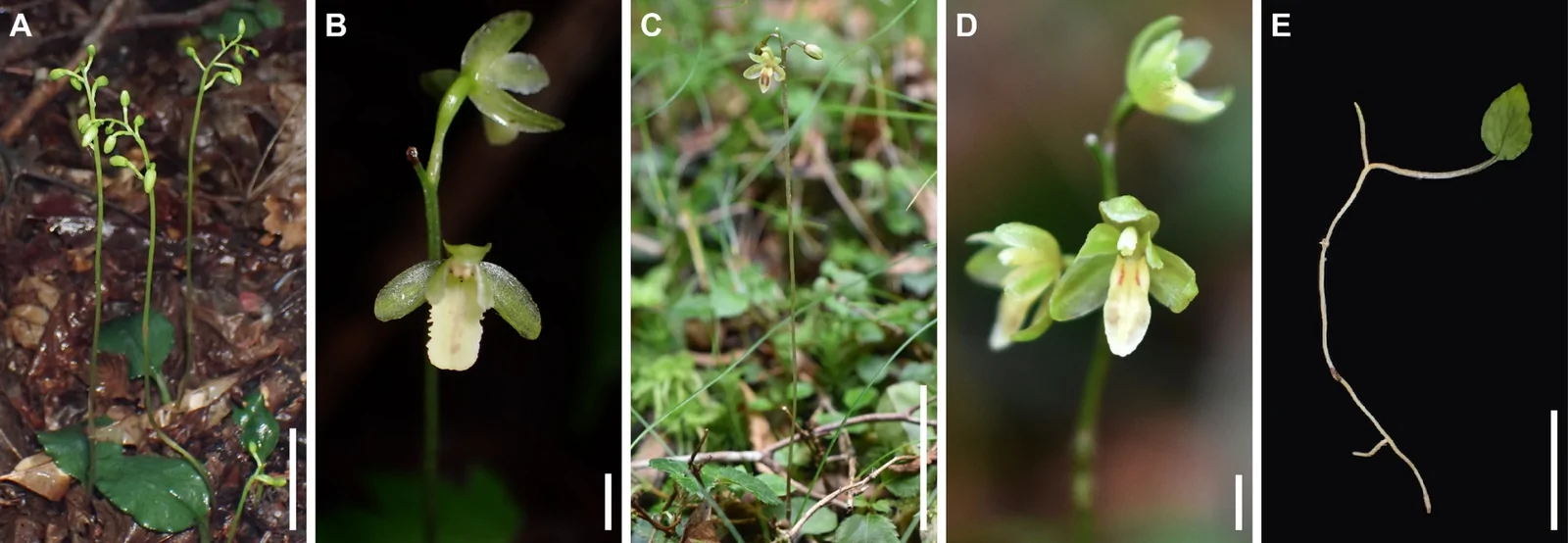
Habit and floral morphology of *Ephippianthus sawadanus* and *E. schmidtii*. (**A**) Flowering plants of *E. sawadanus*. (**B**) Flowers of *E. sawadanus*. (**C**) Flowering plant of *E. schmidtii*. (**D**) Flowers of *E. schmidtii*. (**E**) Growth habit and creeping rhizomes of *E. schmidtii*. Scale bars = 3 cm (**A**, **C**, **E**) and 3 mm (**B**, **D**)

Field investigations for stable isotope analysis and molecular identification of mycorrhizal partners were conducted at one site per species. The population of *Ephippianthus sawadanus* was surveyed at Suyama, Susono City, Shizuoka Prefecture, Japan (Fig. 1), on 29 July 2020 during flowering, when approximately 30 flowering individuals were observed. The site was a cool-temperate montane forest dominated by *Quercus crispula*, *Pieris japonica*, *Acer tschonoskii*, *Acer mono*, *Betula grossa*, and *Abies homolepis*, with a dense understory of *Sasa borealis*. Sampling of *E. schmidtii* was conducted at Awakura, Fujinomiya City, Shizuoka Prefecture, on 15 June 2021, where approximately 100 individuals were present in a subalpine conifer–broadleaf forest dominated by *Tsuga diversifolia*, *Picea jezoensis* var. *hondoensis*, *Alnus matsumurae*, *Sorbus commixta*, and *Pinus parviflora*.

For each species, we established four 1 × 1 m plots at the study site. For stable isotope analysis, one flowering shoot was collected from each plot, yielding four plot-level replicates per species. For molecular identification of fungal partners, rhizome segments were collected from one flowering individual per plot for *E. sawadanus* and from two flowering individuals per plot for *E. schmidtii*. The metabarcoding data were used descriptively to identify the principal fungal associates of each species, rather than to compare fungal community composition among plots. To minimize disturbance, only small rhizome segments were collected, usually one or two pieces approximately 1–2 cm long per individual, from which mycorrhizal fragments were later excised for DNA extraction.

Following Gebauer and Meyer (2003), leaves of co-occurring understory species similar in height to *Ephippianthus* were collected as autotrophic reference plants for δ¹³C and δ¹⁵N analysis. All plant samples were oven-dried at 60 °C and finely ground in an agate mortar before isotope analysis.

### Molecular identification of mycorrhizal fungi

Rhizomes were rinsed to remove adhering soil, and cortical tissues were examined under a compound light microscope to confirm the presence of intracellular hyphal coils, or pelotons, characteristic of orchid mycorrhiza. Mycorrhizal fragments were successfully obtained from three *E. sawadanus* individuals and seven *E. schmidtii* individuals. Excised mycorrhizal fragments, approximately 3 mm long, were surface-sterilized in 1% sodium hypochlorite for 30 s and rinsed three times in sterile distilled water for 30 s each. DNA was extracted from the surface-sterilized tissues using the cetyltrimethylammonium bromide (CTAB) method (Doyle and Doyle 1990).

The fungal ITS2 region was amplified with two primer sets: ITS86F/ITS4, targeting broad orchid mycorrhizal communities (Waud et al. 2014), and a *Tulasnella*-targeting primer pair consisting of the newly designed forward primer 5.8S-Tulngs-7 (5′-CCTTGGCACGTCATTC-3′) and the reverse primer ITS4-Tul2 (Oja et al. 2015). All primers were fused at their 5′ ends with 3–6 random nucleotides and Illumina sequencing primer sequences. PCR was performed using Q5 High-Fidelity DNA Polymerase (New England Biolabs, Ipswich, MA, USA) under the following conditions: 98 °C for 40 s; 35 cycles of 98 °C for 5 s, annealing at 58 °C for ITS86F/ITS4 or 61 °C for 5.8S-Tulngs-7/ITS4-Tul2 for 10 s, and 72 °C for 20 s; followed by 72 °C for 10 min. A 12-cycle second-round PCR was used to attach P5 and P7 adapters and sample-specific indices (Syed et al. 2009). Amplicon libraries were sequenced using either an Element AVITI system with the AVITI 2 × 150 Sequencing Kit Cloudbreak FS Low Output (Element Biosciences, USA) or an Illumina MiSeq system with the MiSeq Reagent Micro Kit v2 (300 cycles; Illumina, USA). Sequence data are available in the NCBI Sequence Read Archive under BioProject accession number PRJNA1439540.

Bioinformatic analyses were conducted using Claident v0.9.2024.06.10 (Tanabe and Toju 2013). After primer trimming, reads were quality-filtered and screened to remove chimeric sequences and index-hopping artifacts using clremovechimev and clremovecontam. Operational taxonomic units (OTUs) were clustered at 97% sequence similarity, and the most abundant sequence within each OTU was selected as the representative. OTUs were taxonomically assigned to the lowest possible rank, and putative orchid mycorrhizal fungi were identified based on previous reports, phylogenetic placement, and occurrence in surface-sterilized mycorrhizal tissues (Dearnaley et al. 2012; Wang et al. 2021). Singletons and OTUs with ≤ 10 total reads were excluded, and sequencing depth was standardized by rarefying each sample to 5,597 reads.

Because Cyphellaceae and Tulasnellaceae were the dominant fungal groups associated with *E. sawadanus* and *E. schmidtii*, respectively, phylogenetic analyses were performed for OTUs in these families and related taxa. Representative OTU sequences were subjected to BLAST searches against the INSDC databases (Altschul et al. 1997), and related sequences were retrieved. Multiple sequence alignments were generated using PRANK (Löytynoja 2021), and maximum-likelihood trees were constructed with IQ-TREE v3.0.1 (Wong et al. 2026). Best-fit substitution models were selected with ModelFinder under the Bayesian Information Criterion (Kalyaanamoorthy et al. 2017). Branch support was assessed using the Shimodaira–Hasegawa-like approximate likelihood ratio test (SH-aLRT) and ultrafast bootstrapping (UFBoot), each with 1,000 replicates (Guindon et al. 2010; Hoang et al. 2018).

### δ¹³C and δ¹⁵N analysis

The natural abundances of ¹³C and ¹⁵N in *E. sawadanus*, *E. schmidtii*, and neighboring autotrophic reference plants were measured using a Delta XP mass spectrometer connected to a Flash EA 2000 elemental analyzer (Thermo Fisher Scientific, Waltham, MA, USA). Relative isotope abundances were expressed as:

δ¹³C or δ¹⁵N = (*R*_sample_/*R*_standard_ – 1) × 1,000 [‰],

where *R*_sample_ represents the ^13^C/^12^C or ^15^ N/^14^N ratio of the sample, and *R*_standard_ represents the corresponding ratio of Vienna Pee Dee Belemnite for carbon or atmospheric N₂ for nitrogen. Carbon and nitrogen isotope ratios were calibrated using laboratory standards CERKU-01, -02, and − 03 (Tayasu et al. 2011). Analytical standard deviations were < 0.09‰ for δ^13^C (*n* = 24) and < 0.22‰ for δ^15^N (*n* = 24). Total C and N concentrations were determined from sample weights and gas concentrations (CO_2_ and N_2_) using the same standards. Enrichment factors (ε^13^C and ε^15^N) were calculated as the differences between the δ^13^C and δ^15^N values of each *Ephippianthus* specimen and the mean δ^13^C and δ^15^N values of autotrophic reference plants from the same plot (Preiss and Gebauer 2008).

Differences in δ¹³C and δ¹⁵N values between each *Ephippianthus* species and its neighboring autotrophic reference plants were tested using linear mixed models, with plant category as a fixed effect and plot as a random effect. Each isotope variable was analyzed separately. Model assumptions were evaluated using Q–Q plots, which showed no obvious deviation from normality. All statistical analyses were performed in R (R Core Team 2025).

## Results

### Molecular identification of mycorrhizal fungi

**Fig. 2:**
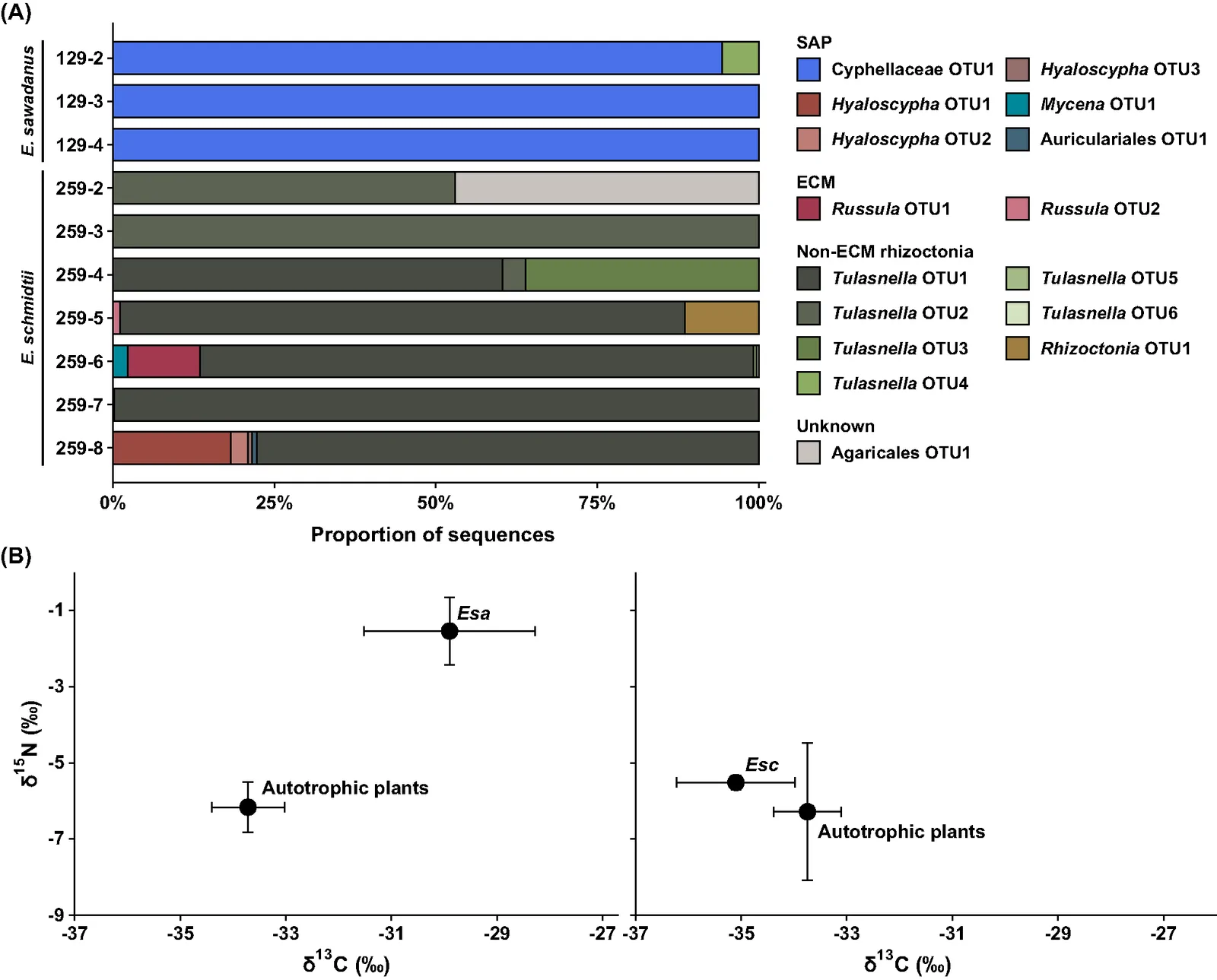
(**A**) Relative abundances of fungal OTUs in mycorrhizal communities associated with the two *Ephippianthus* species. (**B**) Mean (± SD) δ¹³C and δ¹⁵N values for *Ephippianthus sawadanus* and its neighboring autotrophic reference plants (left), and for *E. schmidtii* and its neighboring autotrophic reference plants (right). Sample sizes were *n* = 4 for *E. sawadanus*, *n* = 12 for its autotrophic references, *n* = 4 for *E. schmidtii*, and *n* = 14 for its autotrophic references. *Esa*, *Ephippianthus sawadanus*; *Esc*, *Ephippianthus schmidtii*

In the rarefied dataset, two OTUs and 16,791 reads were recovered from the mycorrhizal rhizomes of three successfully analyzed *E. sawadanus* individuals (Fig. 2A; Table S1). The community was dominated by one Cyphellaceae OTU, which occurred in all three individuals and accounted for 16,471 reads (98.09%). Phylogenetic analysis placed this OTU in a clade with the saprotrophs *Hemimycena mairei* and *H. gracilis* (Fig. S1). However, recent studies show that *Hemimycena* is polyphyletic, with many species traditionally placed in this genus clustering with *Phloeomana* within Cyphellaceae (Vizzini et al. 2022, 2024). Given the unresolved generic boundaries in this group, we conservatively treat the OTU as Cyphellaceae sp. The second OTU, assigned to Tulasnellaceae, was found in only one individual and represented 320 reads (1.91%).

In contrast, *E. schmidtii* was dominated by rhizoctonia fungi in all seven successfully analyzed individuals. Five Tulasnellaceae OTUs accounted for 33,827 reads (86.34%), whereas one *Rhizoctonia* (Ceratobasidiaceae) OTU accounted for 641 reads (1.64%) (Fig. 2A; Table S1). Tulasnellaceae OTUs occurred in all seven individuals, with Tulasnellaceae OTU1 detected in five individuals and contributing 23,003 reads (58.71%). Phylogenetic reconstruction showed that the dominant Tulasnellaceae OTUs associated with *E. schmidtii* clustered with known mycorrhizal partners of other rhizoctonia-associated orchids (Fig. S2). The *Rhizoctonia* OTU detected here did not fall within known ectomycorrhizal clades (Veldre et al. 2013).

Several minor OTUs were also detected in *E. schmidtii*, including unassigned Agaricales OTU1 in two individuals (2,636 reads; 6.73%), *Hyaloscypha* OTU1 in two individuals (1,031 reads; 2.63%), *Hyaloscypha* OTU2 in two individuals (151 reads; 0.39%), *Hyaloscypha* OTU3 in two individuals (39 reads; 0.10%), *Russula* OTU1 (627 reads; 1.60%), *Russula* OTU2 (60 reads; 0.15%), *Mycena* OTU1 (128 reads; 0.33%), and Auriculariales OTU1 (39 reads; 0.10%). Because these non-rhizoctonia OTUs occurred at low relative abundances, they were treated as minor community components.

### δ¹³C and δ¹⁵N analysis

*Ephippianthus sawadanus* exhibited marked ¹³C and ¹⁵N enrichment relative to co-occurring autotrophic plants. The δ¹³C values of *E. sawadanus* (− 29.9 ± 1.6‰, *n* = 4) were significantly higher than those of neighboring autotrophic plants (− 33.7 ± 0.7‰, *n* = 12; *P* < 0.001; Fig. 2B). Likewise, δ¹⁵N values of *E. sawadanus* (− 1.5 ± 0.9‰, *n* = 4) were significantly higher than those of autotrophic references (− 6.2 ± 0.7‰, *n* = 12; *P* < 0.001; Table S2). The enrichment factors ε¹³C and ε¹⁵N were 3.8 ± 1.5‰ and 4.6 ± 1.0‰, respectively.

In contrast, *E. schmidtii* showed no positive ¹³C or ¹⁵N enrichment relative to surrounding autotrophic vegetation. The δ¹³C values of *E. schmidtii* (− 35.1 ± 1.1‰, *n* = 4) were slightly but significantly lower than those of autotrophic plants at the same site (− 33.7 ± 0.6‰, *n* = 14; *P* = 0.006). The δ¹⁵N values of *E. schmidtii* (− 5.5 ± 0.2‰, *n* = 4) did not differ significantly from those of autotrophic plants (− 6.3 ± 1.8‰, *n* = 14; *P* = 0.418; Table S3). The enrichment factors ε¹³C and ε¹⁵N were − 1.4 ± 1.4‰ and 0.7 ± 0.3‰, respectively.

## Discussion

Metabarcoding analyses revealed pronounced differences in the mycorrhizal communities of the two *Ephippianthus* species. The rhizomes of *E. schmidtii* were predominantly colonized by rhizoctonia fungi, which are typical symbionts of photosynthetic orchids. In contrast, *E. sawadanus* was primarily associated with Cyphellaceae (Agaricales), a typically saprotrophic lineage that includes fungi inhabiting decaying wood and leaf litter (Vizzini et al. 2022, 2024). To our knowledge, this is the first documented case in which a member of this family was detected as the dominant mycorrhizal associate of a green orchid.

Stable isotope data supported contrasting nutritional strategies between the two species. *Ephippianthus schmidtii* showed no significant difference from autotrophs in δ¹⁵N values, and its δ¹³C values were slightly but significantly lower. These results provide no evidence of fungal carbon or nitrogen gain detectable by δ¹³C and δ¹⁵N analyses, although cryptic partial mycoheterotrophy remains possible and should be tested using δ²H analysis (Gebauer et al. 2016; Gomes et al. 2023; Zahn et al. 2023). Interestingly, *E. schmidtii* was occasionally associated with fungi such as *Russula* and *Mycena*, genera that include known mycorrhizal partners of highly mycoheterotrophic orchids (Taylor and Bruns 1997; Bidartondo et al. 2004; Ogura-Tsujita et al. 2009). Given their low read abundance and the absence of ¹³C or ¹⁵N enrichment, these fungi likely make little contribution to the adult carbon budget of *E. schmidtii*. Nevertheless, such facultative associations may provide ecological opportunities for shifts toward increased heterotrophy, consistent with the waiting room hypothesis (Selosse et al. 2022).

In contrast, *E. sawadanus* exhibited pronounced ¹³C and ¹⁵N enrichment relative to autotrophic reference plants. Its ¹³C enrichment (3.8 ± 1.5‰) is similar to the mean enrichment reported for partially mycoheterotrophic orchids (3.2‰; *n* = 189; Hynson et al. 2016), indicating a substantial fungal contribution to its carbon budget. Its ¹⁵N enrichment (4.6 ± 1.0‰) is also similar to values reported for partially mycoheterotrophic Calypsoinae species associated with saprotrophic Psathyrellaceae (ca. 4‰; Yagame et al. 2021; Zahn et al. 2022; Suetsugu and Okada 2025a, b). Nevertheless, *E. sawadanus* showed lower ¹⁵N enrichment than the mean reported for orchids associated with ectomycorrhizal fungi (9.6‰; Hynson et al. 2016; Schiebold et al. 2017), likely reflecting the generally lower ¹⁵N enrichment of saprotrophic fungi relative to ectomycorrhizal fungi (Gebauer and Taylor 1999).

Recruitment of saprotrophic fungi has occurred repeatedly during the evolution of fully mycoheterotrophic orchids (Martos et al. 2009; Ogura-Tsujita et al. 2009; Lee et al. 2015; Suetsugu et al. 2020), and evidence from the *Cremastra appendiculata* species complex indicates that such associations can precede the loss of photosynthesis (Suetsugu et al. 2022). The association with Cyphellaceae in *E. sawadanus* provides further evidence that recruitment of saprotrophic fungi into orchid mycorrhizal partnerships may promote increased fungal dependence. Intriguingly, a Cyphellaceae fungus, *Mycopan scabripes*, has also been reported as a symbiont of the fully mycoheterotrophic orchid *Gastrodia flavilabella* (Lee et al. 2015; Vizzini et al. 2022), suggesting that Cyphellaceae fungi can support orchids with substantial fungal carbon acquisition.

Within the *Corallorhiza* clade, increased heterotrophy has repeatedly coincided with shifts to ectomycorrhizal fungi in *Kitigorchis* and *Corallorhiza* or to saprotrophic fungi in *Oreorchis* and *Cremastra* (Barrett et al. 2025). The saprotrophic agaric partner and strong isotopic enrichment of *E. sawadanus*, together with the largely autotrophic status of *E. schmidtii* and *Dactylostalix* (Suetsugu and Okada 2025c), suggest an independent origin of strong partial mycoheterotrophy in the *Dactylostalix*–*Ephippianthus* lineage. Unlike many highly heterotrophic Calypsoinae species (Yagame et al. 2021; Suetsugu et al. 2022; Suetsugu and Okada 2025a), *E. sawadanus* lacks coralloid rhizomes, suggesting that such structures are not essential for substantial fungal carbon acquisition.

In summary, our study reveals pronounced differences in mycorrhizal associations and nutritional strategies between the two known *Ephippianthus* species. *Ephippianthus schmidtii* primarily associates with rhizoctonia fungi and shows no detectable ¹³C or ¹⁵N enrichment relative to autotrophic reference plants. In contrast, *E. sawadanus* is predominantly associated with saprotrophic Cyphellaceae and shows strong ¹³C and ¹⁵N enrichment consistent with partial mycoheterotrophy. These contrasting strategies within a single genus highlight the evolutionary flexibility of orchid–fungus interactions and suggest that recruitment of saprotrophic fungal partners can promote increased fungal dependence. However, because each species was sampled from a single population, broader surveys are needed to determine whether these fungal associations and isotope signatures are geographically stable.

## Supplementary Information

Below is the link to the electronic supplementary material.

Supplementary Material 1 (XLSX 26.4 KB)

Supplementary Material 2 (PDF 194 KB)

## Data Availability

Sequence data have been deposited in the NCBI Sequence Read Archive under BioProject accession number PRJNA1439540.
