# Supplementary material for: Divergent mycorrhizal strategies and nutritional modes in the East Asian orchid genus Ephippianthus
Source: Mycorrhiza. 2026 Jul 2;36(4):45. doi: 10.1007/s00572-026-01285-0 (PMC13323335; doi:10.1007/s00572-026-01285-0)
Supplement: Supplementary file 2 — Supplementary Material 2 (PDF 194 KB) [file 572_2026_1285_MOESM2_ESM.pdf]

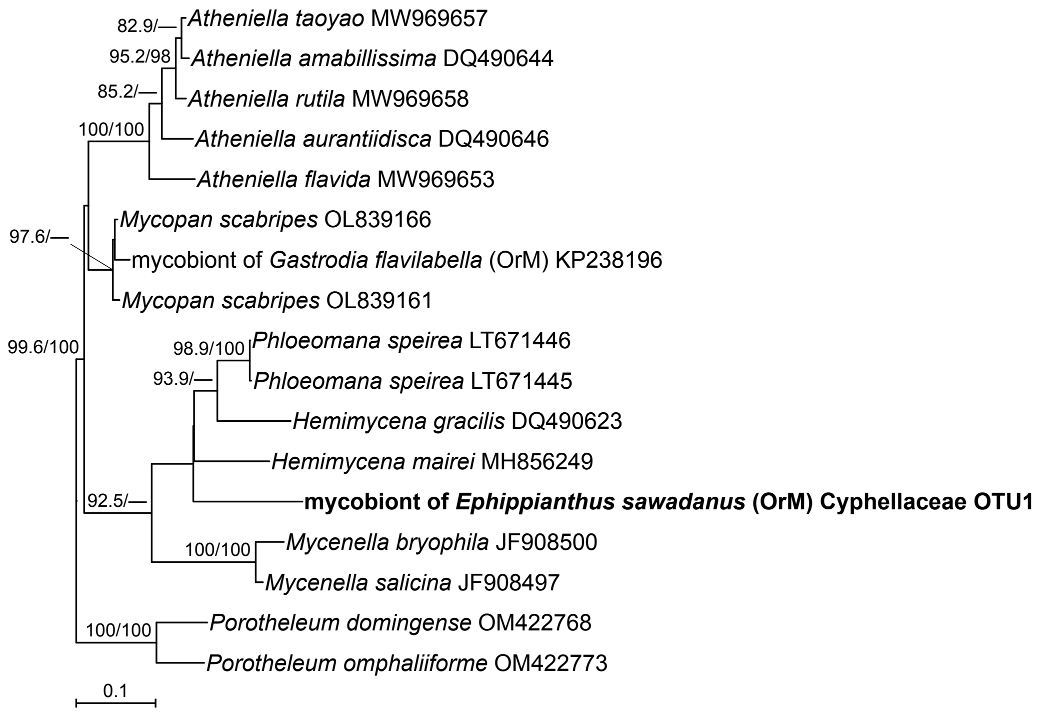

Fig. S1. Maximum-likelihood phylogenetic tree based on ITS2 rDNA sequences of the Cyphellaceae operational taxonomic unit (OTU) detected in mycorrhizal samples of *Ephippianthus sawadanus* (shown in bold), together with related sequences retrieved from the INSDC databases. Accession numbers are indicated for all reference sequences. The tree was rooted using *Porotheleum domingense* and *P. omphaliiforme* (Porotheleaceae) as outgroups. Node labels indicate SH-aLRT/ultrafast bootstrap support; values below 80% and 95%, respectively, are replaced by an em dash. The scale bar indicates substitutions per site. Abbreviation: OrM, orchid mycorrhizal fungi.

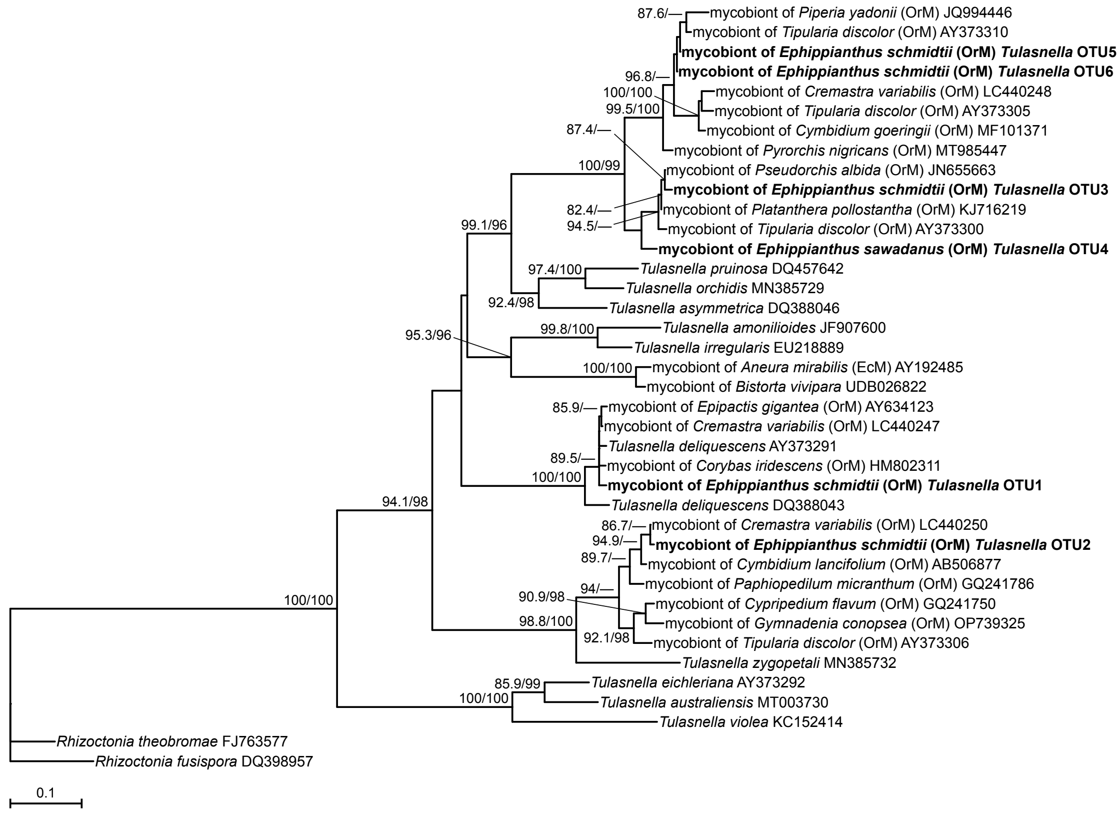

Fig. S2. Maximum-likelihood phylogenetic tree based on ITS2 rDNA sequences of the Tulasnellaceae operational taxonomic units (OTUs) detected in mycorrhizal samples of *Ephippianthus schmidtii* and *E. sawadanus* (shown in bold), together with related sequences retrieved from the UNITE and INSDC databases. Accession numbers are indicated for all reference sequences. The tree was rooted using *Rhizoctonia theobromae* and *R. fusispora* (Ceratobasidiaceae) as outgroups. Node labels indicate SH-aLRT/ultrafast bootstrap support; values below 80% and 95%, respectively, are replaced by an em dash. The scale bar indicates substitutions per site. Abbreviations: OrM, orchid mycorrhizal fungi; EcM, ectomycorrhizal fungi.
